# Successful surgical treatment of a giant coronary artery aneurysm presenting with recurrent profuse haemoptysis

**DOI:** 10.1186/1749-8090-3-36

**Published:** 2008-06-29

**Authors:** Opoku-Ware Mensah, Philip AR Hayward, Michael Koeppe, Christof Huth

**Affiliations:** 1 Essex Cardiothoracic Centre, Basildon, UK; 2 Klinik für Herz- und Thoraxchirurgie der Otto-von-Guericke-Universität Magdeburg, Germany

## Abstract

We present the case of successful resection of a giant aneurysm of the LAD presenting with recurrent severe haemoptysis in a 72-year old man. He was admitted to a regional hospital with fever, recurrent bloody sputum, weight loss and left sided chest pain, and developed respiratory failure requiring ventilation. Investigations are summarised and reviewed and the diagnosis was eventually reached by TTE, CT and MRI scans, confirmed by coronary angiography. Successful emergency surgery to resect the aneurysm and put a vein graft to the LAD is described. The presentation and management of coronary giant aneurysm is reviewed.

## Introduction

Coronary artery aneurysm is a clinical entity, which is found rarely during coronary angiography. Most patients have typical coronary artery disease symptoms. We present here a case of a giant coronary artery aneurysm of the LAD presenting with recurrent profuse haemoptysis.

## Case presentation

**Figure 1:**
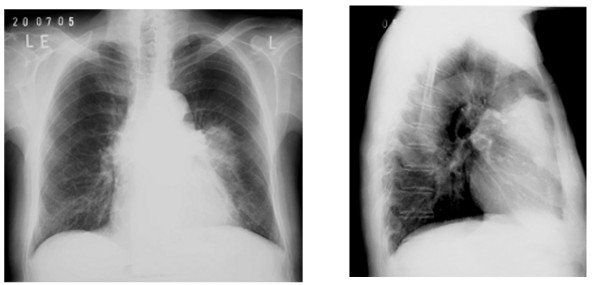
Chest x-ray in anterior-posterior projections showing an anterior-medial infiltrate of the left lung.

**Figure 2:**
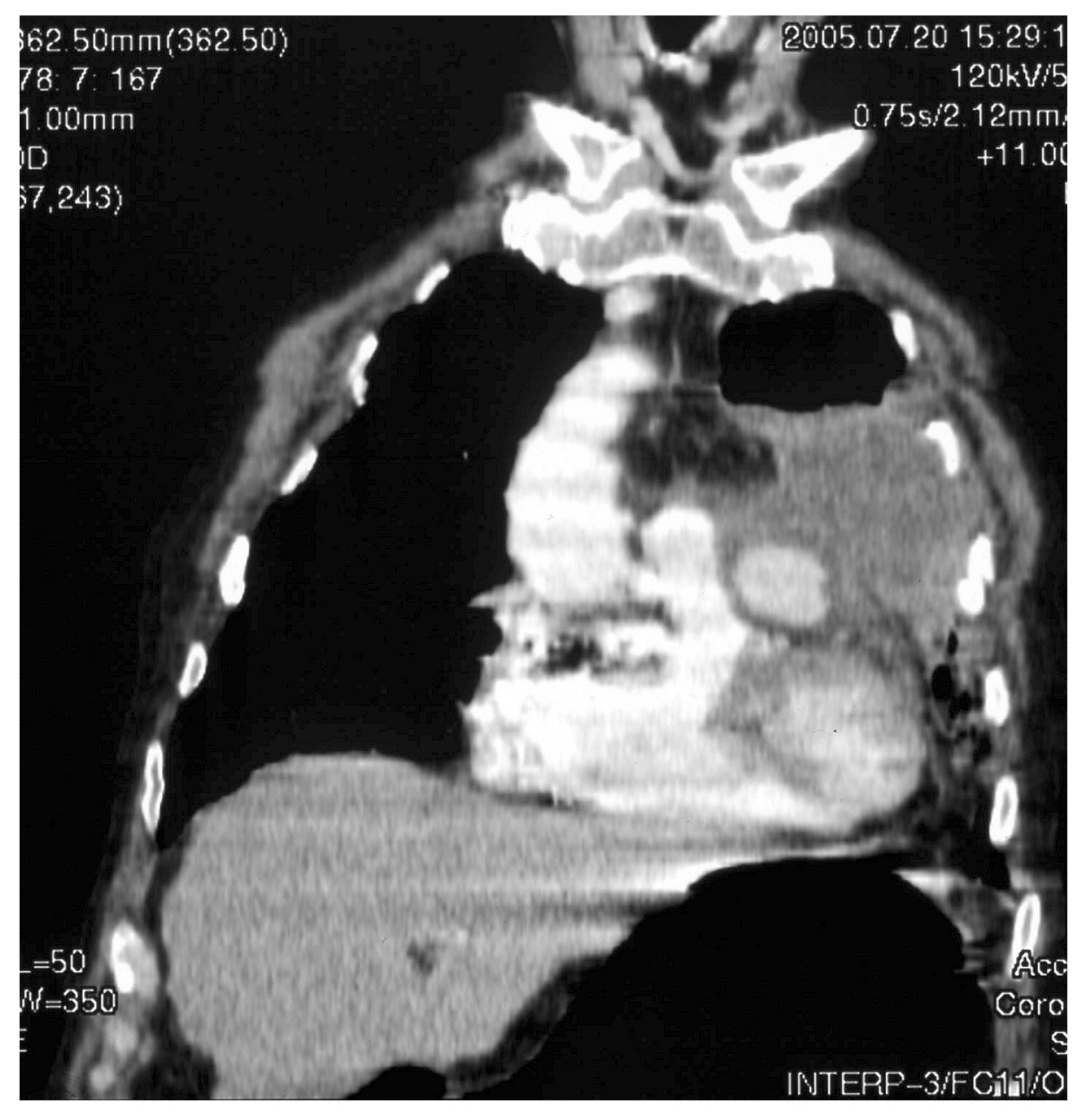
Preoperative MRT showing the coronary artery (LAD) aneurysm and infiltration of the upper lobe.

**Figure 3:**
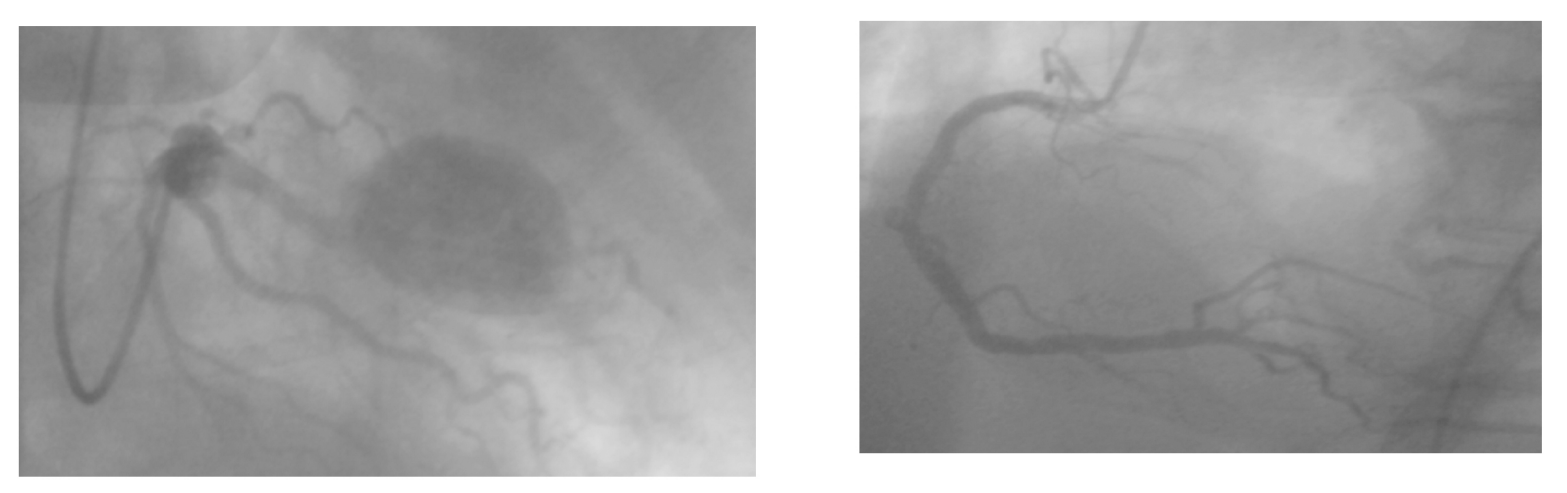
The coronary angiography showing the giant coronary artery aneurysm of the LAD and a normal right coronary artery.

A 72-year old man, previously well, was admitted to a regional hospital with fever, recurrent bloody sputum, weight loss and left sided chest pain of 4 weeks duration. A chest radiograph showed a mass at the left hilum thought to be a neoplasm (see figure 1). A misdiagnosis of haematemesis led to an upper gastrointestinal endoscopy showing oesophagitis. He developed respiratory failure requiring ventilation. A subsequent bronchoscopy excluded a central bronchial tumour, confirmed by histopathologic examination of biopsies. He was transferred to a specialist centre where a CT and MRT scans of the thorax showed the mass to be infiltrating the left upper lobe and was suspicious for an aneurysm of a coronary artery (see figure 2). Transthoracic echocardiography and MRI confirmed this impression and he proceeded to coronary angiography, which demonstrated a giant aneurysm from the LAD (figure 3). He developed cardiovascular instability and proceeded emergently to surgery.

**Figure 4:**
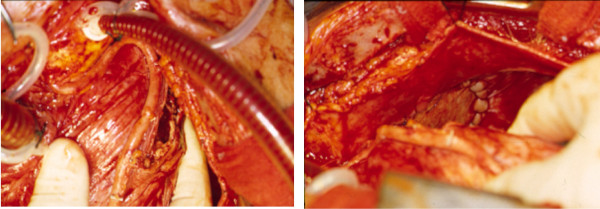
Intra-operative images showing the aortacoronary vein bypass to the LAD, as well as the Situs after the Peri-Guard Patch closure of the pericardial defect resulting from resection of the coronary artery aneurysm.

The procedure was performed using right atrial to aortic cardiopulmonary bypass via median sternotomy and opening of the anterior pericardium. The aneurysm was adherent to the left anterolateral pericardial aspect and was not disturbed until cardioplegic arrest was achieved, to avoid distal embolization in a beating heart. After achieving myocardial arrest the dissection was completed, revealing the aneurysm to have entry and exit openings into the proximal and mid LAD (see figure 4), and there was compression of the left upper lobe with airway inflammation rather than invasion. The adherent pericardium was removed together with the aneurysm and a large quantity of thrombus, leaving a significant pericardial defect requiring Supple-Guard patch closure. The proximal and distal communications from the LAD to aneurysm were closed. A single end-to-side vein graft was performed in the mid LAD distal to the aneurysm (see figure 4). After reperfusion and rewarming cardiopulmonary bypass was weaned without difficulty. Thereafter the medial surface of the left upper lobe was inspected, confirming absence of a fistula.

The patient was transferred to the Intensive Care Unit where after a period of inotropic support, he was stabilised and extubated on the 2^nd ^post-operative day but developed respiratory insufficiency requiring 5 further days of ventilation. He was discharged well on the 27^th ^post-operative day and had no further events at follow up to 7 months.

## Discussion

Coronary artery aneurysm is found in only about 0.15% to 4.9% of patients who undergo coronary angiography [1]. Aneurysmal diameter varies from 1.5 – 2 × that of the largest coronary artery, up to the so-called giant aneurysm with a diameter of 5 cm to 15 cm [2,3]. Its aetiology may be congenital, or acquired through atherosclerosis, Kawasaki disease, complications of percutaneous coronary artery angioplasty, chest trauma [4] and rarely cocaine abuse [5]. There is geographic variation with atherosclerotic aneurysm found mainly in patients of European origin, whereas the giant aneurysm found characteristically in Kawasaki disease occurs predominantly in Japanese, Chinese and other Asian ancestries.

The gold standard diagnostic imaging procedure is coronary angiography to confirm the origin from a coronary vessel, its size and fistulous communication to other structures. In most cases the diagnosis is already suspected following CT or MRI scanning performed to define a mass lesion, which may suggest a vascular aneurysm in proximity to the epicardial surface as in our patient.

Therapeutic options include surgery or percutaneous coronary stenting combined with adjuvant antiplatelet and anticoagulant agents. Median sternotomy and hypothermic cardiopulmonary bypass are usually utilised. In cases where there is a very large aneurysm with extensive pericardial adhesions, peripheral cannulation for institution of bypass and cooling prior to opening the chest may be prudent [1]. In the case presented, we were confident to perform sternotomy and to free the anterior portion of the ascending aorta and the right atrium for standard cannulation because the aneurysm was clearly lateral to these structures and could be left undisturbed until the aorta had been clamped. Our rational for this approach was to avoid any catastrophic bleeding as well as distal coronary artery embolization. We did not do any extensive dissection of the left upper lobe in order to avoid any injury to the lung.

There have been case reports presenting various intra operative findings such as fistulae from the aneurysm to a cardiac chamber [3]. In the case presented here, we did not find any clear fistula on the basis of the coronary angiography nor in the CT and MRI. In the bronchoscopy no definitive bleeding source could be found. During the operation we could not find any direct vascular connection between the left upper lobe and the aneurysm and the haemoptysis resulted from bronchial bleeding points which, in our opinion, resulted from the compression exerted by the giant aneurysm through the pericardial wall. The absence of any bronchial tumor in the bronchoscopy as well as the bleeding from the ulcerated gastrooesphageal junction observed at gastroscopy did blur the clinical picture and delayed the definitive diagnosis.

## Conclusion

The case presented had a protracted preoperative diagnostic course which may have resulted in life threatening haemorrhage or rupture prior to surgical intervention. Transthoracic echocardiography, chest CT, MRI and coronary angiography were of assistance in arriving at a final and correct diagnosis. We would encourage consideration of a possible intrathoracic aneurysm, for example a giant coronary artery aneurysm, although it is rare, in a patient with haemoptysis and an abnormal mediastinal contour in the presence of normal bronchoscopy.

## Authors' contributions

CH, OWM qualifying the patient for emergency operation; were involved in pre-operative decision-making. CH, OWM and MK participated in the surgical management, OWM and MK were actively involved in the postoperative care and later follow up, OWM prepared and wrote the manuscript. PAH participated in the writing of the manuscript.

## Consent

Written informed consent was obtained from the patient for publication of this case report and accompanying images.
